# Large herbivore assemblages in a changing climate: incorporating water dependence and thermoregulation

**DOI:** 10.1111/ele.13350

**Published:** 2019-07-22

**Authors:** M. P. Veldhuis, E. S. Kihwele, J. P. G. M. Cromsigt, J. O. Ogutu, J. G. C. Hopcraft, N. Owen‐Smith, H. Olff

**Affiliations:** ^1^ University of Groningen Nijenborg 7 9747AG Groningen The Netherlands; ^2^ Princeton University 106A Guyot Ln Princeton NJ 08544 USA; ^3^ Leiden University Einsteinweg 2 2333CC Leiden The Netherlands; ^4^ Tanzania National Parks Arusha Tanzania; ^5^ Department of Wildlife, Fish and Environmental Studies Swedish University of Agricultural Sciences Umeå 901 83 Sweden; ^6^ Centre for African Conservation Ecology, Department of Zoology Nelson Mandela University PO Box 77000 Port Elizabeth 6031 South Africa; ^7^ Environmental Sciences group Copernicus Institute of Sustainable Development, Utrecht University PO Box 80115 3508 TC Utrecht The Netherlands; ^8^ University of Hohenheim, Institute of Crop Science, Biostatistics Unit Fruwirthstrasse 23 70599 Stuttgart Germany; ^9^ University of Glasgow Glasgow G128QQ UK; ^10^ Centre for African Ecology, School of Animal, Plant and Environmental Sciences University of the Witwatersrand Wits 2050 South Africa

**Keywords:** Climate change, hyperthermia, niche differentiation, predation risk, ungulates, water requirements

## Abstract

The coexistence of different species of large herbivores (ungulates) in grasslands and savannas has fascinated ecologists for decades. However, changes in climate, land‐use and trophic structure of ecosystems increasingly jeopardise the persistence of such diverse assemblages. Body size has been used successfully to explain ungulate niche differentiation with regard to food requirements and predation sensitivity. But this single trait axis insufficiently captures interspecific differences in water requirements and thermoregulatory capacity and thus sensitivity to climate change. Here, we develop a two‐dimensional trait space of body size and minimum dung moisture content that characterises the combined food and water requirements of large herbivores. From this, we predict that increased spatial homogeneity in water availability in drylands reduces the number of ungulate species that will coexist. But we also predict that extreme droughts will cause the larger, water‐dependent grazers as wildebeest, zebra and buffalo–dominant species in savanna ecosystems – to be replaced by smaller, less water‐dependent species. Subsequently, we explore how other constraints such as predation risk and thermoregulation are connected to this two‐dimensional framework. Our novel framework integrates multiple simultaneous stressors for herbivores and yields an extensive set of testable hypotheses about the expected changes in large herbivore community composition following climate change.

## Introduction

Predicting how climate change will affect ungulate communities is now urgent (Speakman & Król 2010; Fuller *et al.* 2014; Shrestha *et al.* 2014; Fuller *et al.* 2016; Pigeon *et al.* 2016) because increasing land temperatures, changing rainfall regimes (Niang *et al.* 2014) and habitat fragmentation increase the risk of regional extinctions (Ripple *et al.* 2015). Herbivores thus face rapid changes in the availability of food and water simultaneously. Furthermore, the capacity of species to adapt to these changing resource availabilities will interact with changes in other constraints, such as temperature and predation risk. For effective conservation strategies, we need integrated predictive frameworks that incorporate all of these key determinants of herbivore assemblages. Here, we propose to integrate these constraints for ungulates in grasslands and savannas through a limited set of key functional traits (see Glossary) using the large herbivore assemblages in African savanna ecosystems as a generalisable example. This trait‐based approach aims to capture the main axes of variation with regard to physiology, ecology and evolutionary history (Cadotte *et al.* 2013) into a broader framework. This yields five testable hypotheses (H1‐H5) about changes in ungulate assemblages in response to climate change or management interventions, such as protected area enlargement (including longer landscape gradients), homogenisation of landscape water availability through establishment of artificial water points (e.g. dams for watering livestock), or extirpation or reintroduction of predators.

## Niche partitioning among ungulates: the role of body size

The diversity of mammals in African savannas has intrigued ecologists for decades, particularly the coexistence of so many ungulates that apparently eat similar food. Multiple key insights on dietary niche partitioning have followed since. First, predictable dietary variation is found along the grazer–browser continuum (Lamprey 1963), a separation which has recently been studied in greater detail using differences in isotopic signals of C4 grasses and C3 trees and forbs (Ambrose & Deniro 1986; Cerling *et al.* 2003; Codron *et al.* 2007), or even to the species level using DNA‐barcoding techniques (Kartzinel *et al.* 2015). Second, digestive strategy (ruminant vs. non‐ruminant) and body size capture the trade‐off between foraging on large amounts of low‐quality food (such as including a high proportion of stems and twigs) vs. small amounts of high‐quality food such as young leaves (Illius & Gordon 1992; Wilmshurst *et al.* 2000). Body size variation is therefore commonly used to explain niche differentiation and coexistence along major landscape gradients of plant available moisture and nutrients that together determine the availability and digestive quality of plant biomass (Olff *et al.* 2002; Hopcraft *et al.* 2010). In addition, body size predicts how vulnerable animals are to predation (larger species are generally less vulnerable) (Sinclair *et al.* 2003). This has yielded an established framework for explaining resource partitioning based on interspecific differences in body size and feeding style (grazer–browser continuum) (Olff *et al.* 2002; Gordon & Prins 2008; Hopcraft *et al.* 2010). Based on this framework we expect larger herbivores to be more affected by drought through reduced availability of forage (Olff *et al.* 2002; Hopcraft *et al.* 2010). Furthermore, grazers are expected to be more susceptible to droughts than browsers (Kay 1997; Gordon & Prins 2008). This is because shallow‐rooting grasses dry out much faster with the onset of the dry season than deeper‐rooting woody species. However, this framework is incomplete, as it does not incorporate key components of physiological tolerance of the ecological niche: thermoregulation capacity and water requirements. Given the current rate of climate change, we need to understand if important interspecific differences in adaptations to drought and high temperatures can also be explained by variation in body size, or whether other (independent) functional traits are required to predict species responses to landscape gradients and climate change scenarios. Such an integrated framework will be useful for the design of novel experiments to test underlying mechanisms and to improve predictions of future changes in large herbivores community assembly.

## Thermal tolerance of different‐sized species

**Figure 1:**
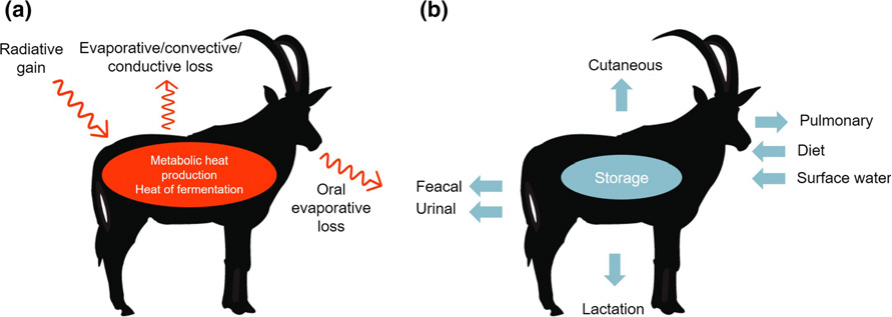
Overview of the primary components of the thermoregulation (a) and water balance (b) of terrestrial endothermic ungulates. Red arrows represent routes of heat gain and loss over a time interval while blue arrows represent water loss and gain (affected by morphology, physiology and behaviour). (a) Heat gain can be reduced by either avoiding direct sunlight or decreased activity, while heat loss can be increased through transpiration, direct contact with cool substrates or increased air flow across the skin. (b) Regular dependence on surface water is lower in species with less water losses or higher dietary water content. Intervals between water intake (surface or dietary) are higher in species with higher internal storage.

Below‐optimal body temperatures potentially restrict the metabolic rate and activity of animals (Gillooly *et al.* 2001; Savage *et al.* 2004). Endotherms can generally maintain high metabolic rates and associated activity despite low external temperatures through homeostasis of body temperature. However, much less known and studied are the negative effects of above‐optimal temperatures in endotherms that can potentially lead to hyperthermia (Speakman & Król 2010; Payne & Bro‐Jørgensen 2016). Body mass is an important determinant of heat balance in endotherms, because larger species have less surface area per unit volume or weight (Porter & Kearney 2009). This causes large animals to more easily retain heat under cold conditions but also to more difficulty loose heat under warm conditions. Problems with loosing heat may thus limit the activity of large ungulate species, as buffalo (*Syncerus caffer*), hippo (*Hippopotamus amphibous*) or elephant (*Loxodonta africana*), under very hot conditions (blue arrows Fig. 1a). Current evidence confirms these predictions and shows that larger ungulates indeed limit their activity more strongly at high temperatures (Du Toit & Yetman 2005; Aublet *et al.* 2009; Gardner *et al.* 2011; Owen‐Smith & Goodall 2014). Moreover, there is evidence that larger animals rely more on sweating and wallowing than small species as an way of losing heat (Robertshaw & Taylor 1969; Parker & Robbins 1984). Lastly, smaller animals also can better create or access burrows, holes, caves and shaded habitats under taller grass, shrubs and trees, all cool microhabitats that allow them to temporarily escape hot times with high solar radiation (Fuller *et al.* 2016). Therefore, body size is a key functional trait for understanding not only the food requirements and predation risk of savanna ungulates, but also for understanding their thermoregulatory constraints.

## Capturing surface water dependence in a key trait

Weak dependence on surface water is beneficial for savanna ungulates as it reduces various costs associated with drinking (Cain *et al.* 2012). For example, it opens up additional foraging areas far away from water, reduces energy costs associated with travel to and from water, enables spatial partitioning with other (more water‐dependent) ungulates for food and reduces exposure to predation (see below). Increasing availability of census data and technical and statistical methodologies have therefore produced a range of new results on how ungulate behaviour is driven by spatio‐temporal availability of surface water, using distance to surface water as a proxy for surface water dependence (Redfern *et al.* 2003; Smit *et al.* 2007; Ogutu *et al.* 2010, 2014; Smit 2011; Cain *et al.* 2012). However, it is still difficult to draw clear general conclusions from these studies due to confounding of water requirements, food requirements and predation risk sensitivity as a the key driver. A more reliable, and more easy to measure, indicator of surface water dependence may instead be found in specific functional traits related to water balance adaptations ([Link] *et al.* Submitted).

Water scarcity in savannas has led to specific morphological, physiological and behavioural adaptations in ungulates, allowing them to survive through the dry season (McNab 2002; Cain *et al.* 2006; Fuller *et al.* 2016; Abraham *et al.* 2019). Reduced dependence on surface water has evolved in ungulates through different adaptations: (1) increasing dietary water intake, (2) higher water storage in the body (also in carbohydrates, proteins or fat for later release as metabolic water), or (3) by reducing water losses (Fig. 1b) (Rymer *et al.* 2016).

During the dry season, the leaf water content of grasses that die off aboveground is generally lower than that of woody plants that remain green. The resulting higher dietary water intake makes browsers generally less surface water‐dependent than grazers (Kay 1997). This dietary water intake can even yield sufficient water for some species to survive for long periods without drinking. Metabolic water production (e.g. through metabolising carbohydrates, fat or proteins that were stored in the body in the wet season) is crucial for dryland granivorous birds and small mammals (Schmidt‐Nielsen 1964; Degen 1997), but seems to play only a small role in the water balance of dryland ungulates (Taylor 1968a).

**Figure 2:**
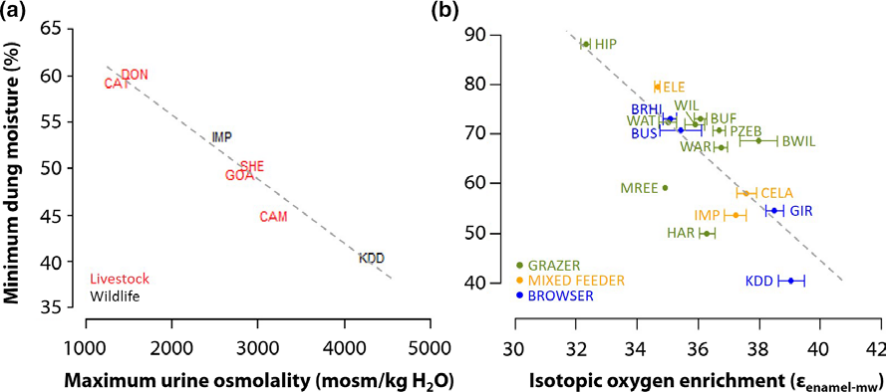
Relationships between key functional traits related to water requirements of savanna ungulates. (a) Negative correlation between minimum dung moisture content and maximum urine osmolality for 6 ungulate species (Linear model: *F*_1,5_ = 128, *P* < 0.001, *R* ^2^ = 0.96). (b) Negative correlation between minimum dung moisture content and isotopic oxygen enrichment for African folivorous ungulates (excluding species with high percentage fruit in their diet because fruits are not enriched in oxygen isotopes) (Linear model: *F*_1,14_ = 20.7, *P* < 0.001, *R* ^2^ = 0.60). Higher levels of enrichment indicate a higher percentage of water derived from food. Abbreviations: BRHI = black rhino, BUF = buffalo, BUS = bushbuck, BWIL = black wildebeest, CAM = camel, CAT = zebu cattle, CELA = common eland, DON = donkey, ELE = elephant, GIR = giraffe, GOA = goat, HAR = hartebeest, HIP = hippopotamus, IMP = impala, KDD = Kirk's dikdik, MREE = mountain reedbuck, PZEB = plains zebra, SHE = sheep, WAR = common warthog, WAT = waterbuck, WIL = common wildebeest. Dung moisture data obtained from (Clemens & Maloiy 1982; King 1983; Maloiy *et al.* 1988; Edwards 1991; Woodall & Skinner 1993; Woodall *et al.* 1999; De Leeuw *et al.* 2001; Sitters *et al.* 2014). Isotopic oxygen enrichment data from (Blumenthal *et al.* 2017). Urine osmolality data from (King 1983). See Online Supplemental information Table S1 for the scientific names of the species.

Ungulates lose water through five routes: pulmonary evaporation, cutaneous evaporation, faeces, urine and lactation ((Rymer *et al.* 2016); Fig. 1b). Faecal and urinary water losses have been studied most extensively in dehydration experiments (Taylor 1968b; Brobst & Bayly 1982) and dissections to study internal organs (Clemens & Maloiy 1982; Woodall & Skinner 1993). These studies show that ungulates exhibit two main physiological adaptations for reducing water loss. Arid adapted species have (1) a relatively long large intestine and smaller circumference of the spiral colon that allows them to resorb more moisture from their faeces (Woodall & Skinner 1993; Woodall 1995) and (2) increased length of the loop of Henlé in the kidney nephron that makes them capable of producing more concentrated urine (Louw & Seely 1982; McNab 2002; Ouajd & Kamel 2009). These traits are phylogenetically correlated: species that typically produce dry dung can also produce highly concentrated urine (Kihwele *et al.* Submitted; Louw & Seely 1982) (Fig. 2a; LM: *F*_1,5_ = 128, *R* ^2^ = 0.96, *P* < 0.001). Selective pressures for one mode of water conservation will also likely favor the other. It can therefore be expected that across species, traits that restrict pulmonary and cutaneous water losses are correlated with traits that restrict faecal and urinary water loss (Kihwele *et al.* Submitted). For example, species that need to obtain most water from drinking surface water produce relatively wet dung. In contrast, very dry dung pellets are produced by species that obtain a substantial proportion of their water from leaves, as demonstrated through oxygen isotope enrichment (Kohn 1996; Blumenthal *et al.* 2017) (Fig. 2b; Linear model: *F*_1,14_ = 20.7, *R* ^2^ = 0.71, *P* < 0.001). The strong correlations between these three traits (dung moisture, urine osmolality, isotopic oxygen enrichment) likely reflect physiological niche differentiation among species along landscape aridity gradients. Overall, this strongly suggests that the capacity to resorb water from dung (minimum dung moisture) and urine (maximum urine osmolality) are reliable indicators of the water requirements of ungulates (Kihwele *et al.* Submitted).

## The interplay of food and water requirements

**Figure 3:**
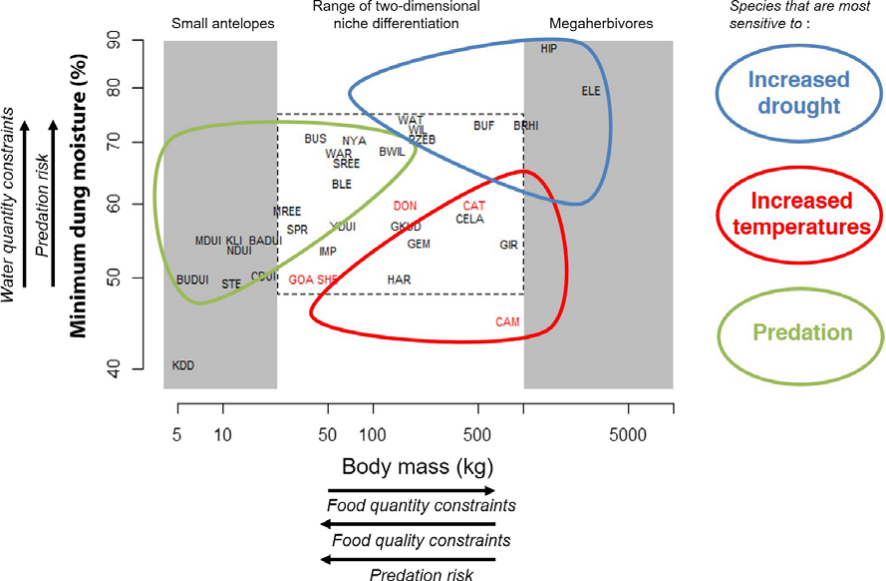
Predicted consequences of environmental change (temperatures, rainfall, predator abundance) for savanna ungulates across the food and water requirements dimensions, based on the outlined interactions between food quantity and quality and water requirements of different species. Within the intermediate range of 20–1000 kg of body mass there is a full occupation of niche space in both dimensions. Global change is expected to affect larger ungulates more strongly but increasing temperatures and droughts have opposing effects between water‐dependent and independent species. The addition of a second dimension suggests a trade‐off between thermal stress and exposure to predation. In addition to abbreviations in Fig. 2: BADUI = bay duiker, BLE = blesbok, BUDUI = blue duiker, CDUI = common duiker, GEM = gemsbok, GKUD = greater kudu, KLI = klipspringer, MDUI = Maxwell's duiker, NDUI = natal duiker, NYA = nyala, SPR = springbok, SREE = southern reedbuck, STE = steenbok. Livestock species are shown in red. Mean female body mass data from (Smith *et al.* 2003; Kingdon *et al.* 2013). Dung moisture data obtained from (Clemens & Maloiy 1982; King 1983; Maloiy *et al.* 1988; Edwards 1991; Woodall & Skinner 1993; Woodall *et al.* 1999; De Leeuw *et al.* 2001; Sitters *et al.* 2014). See Online Supplemental information Table S1 for the scientific names of the species and their body sizes.

To study the interdependence between food and water constraints we explore the relation between body mass (capturing food requirements) and minimum dung moisture content (capturing water requirements) using published data sets. Minimum dung moisture increases with body size (Linear model: *F*_1,33_ = 17.2, *R* ^2^ = 0.34, *P* < 0.001; Fig. 3) but this relation is especially determined by the largest and smallest species. Megaherbivores (> 1000 kg) have high dung moisture contents of 70–90%. In contrast, small ungulates (< 20 kg) have low dung moisture contents. Excluding these largest and smallest species, the relationship between body mass and dung moisture content disappears (Linear model: *F*_1,22_ = 0.003, *R* ^2^ = 0.0001, *P* = 0.95; dashed square Fig. 3). Surprisingly, grazers and browsers do not have different minimum dung moisture content (anova: *F*_2,19_ = 1.59, *R* ^2^ = 0.05, *P* = 0.22), suggesting that water requirements do not differ between grazers and browsers (in contrast to surface water dependence due to differences in dietary water intake), but this remains to be tested through quantifying minimum fundamental frequency of drinking (Cain *et al.* 2012). Dung moisture was higher for non‐ruminants (anova: *F*_1,21_ = 12.2, *R* ^2^ = 0.37, *P* = 0.0.02), suggesting decreased water dependence for ruminants which is in agreement with the finding that artiodactyls evolved and speciated under arid conditions (Strauss *et al.* 2017). Species generally classified as surface water‐independent (Western 1975; Woodall & Skinner 1993; Kingdon *et al.* 2013) such as (from small to large), springbok (*Antidorcas marsupialis*), hartebeest (*Alcelaphus buselaphus*), gemsbok (*Oryx gazella*), camel (*Camelus dromedarius*) and giraffe all have low dung moisture contents while species classified as water‐bound like southern reedbuck (*Redunca arundinum*), common warthog (*Phacochoerus africanus*), common wildebeest (*Connochaetes taurinus*), plains zebra (*Equus quagga*) and African buffalo have high dung moisture contents (Western 1975; Woodall & Skinner 1993; Kingdon *et al.* 2013). This large range in body size for both water‐dependent and water‐independent species, as also shown in Fig. 3, suggests the existence of an additional axis of niche differentiation that is independent of body size. We therefore suggest two main dimensions for niche differentiation in savanna ungulates, related to forage (Box 1) and surface water availability (Box 2), respectively. The addition of this second dimension allows us to understand how similar‐sized grazers or browsers can co‐exist in the same ecosystem by using habitats characterised by different distance to surface water. From this, we predict that *increased spatial homogeneity in surface water availability (water sources everywhere in the landscape, such as artificial water points or dams for watering livestock that increase everywhere across arid Africa) reduces the number of ungulate species that can coexist* (**H1**). Furthermore, we expect that *extreme droughts will have the most negative impact on the largest grazers that depend most on surface water* (**H2**; Fig. 3). We now continue to discuss how other constraints, such as thermoregulation and predation risk might play out across this two‐dimensional framework.

Box 1 **Changes in livestock species composition**

Rangelands in semi‐arid parts of Africa are often degraded, as indicated by reduced herbaceous vegetation cover, increased exposure of bare soil and loss of productivity (Milton *et al.* 1994). This degradation results from multiple causes, including climatic extremes (Cai *et al.* 2014) and livestock overgrazing (Ayoub 1998). As such, rangeland degradation in recent decades could be viewed as representing an extreme ecosystem state that protected areas could approach under climate change, where elevated stress (both abiotic and biotic) has resulted in landscapes with limited forage and water retention capacity (Snyman 2005), i.e. drought. Recent studies show significant decreases in cattle (*Bos taurus indicus*) population size in Kenya's rangelands of approximately 25% between 1977–1980 and 2011–2013 (Ogutu *et al.* 2016). Cattle are slowly being replaced by sheep (*Ovis aries*) and goats (*Capra hircus*) that increased by 76.3% in the same period and, to a lesser extent, by camel (*Camelus dromedarius*, 13.1%) and donkey (*Equus asinus*, 6.7%). This pattern is consistent with the prediction that the ratio of cattle to sheep and goats should decrease with increasing aridity in Kenya's rangelands (Peden 1987). The increasing species are better able to survive extended periods of drought and can graze shorter grass better than cattle or switch to browsing so that they are still able to forage in dry areas or periods. Also, these species (sheep, goats and camel) have generally drier dung (Fig. 3), suggesting that they are better able to resorb water from their dung. We thus expect a shift towards species with low minimum dung moisture in wild ungulate assemblages with increasing droughts and generally more mixed‐feeders and/or browsers. Increased rainfall variability could amplify such shifts because rainfall is the most critical climatic component for ungulates in savannas. Rainfall governs ungulate biomass, population dynamics and distribution through its controlling influence on surface water distribution, forage production and quality (Western 1975; East 1984). Greater rainfall variability would thus exert stronger controls on ungulate population dynamics in savannas, through its influence on calving rates and deaths during severe droughts, especially of breeding females and immature animals (Angassa & Oba 2007).

## Trade‐offs between thermoregulatory and food requirements

Ungulates not only need to balance foraging and drinking but simultaneously face thermoregulatory challenges. Too low or too high temperatures can force them to seek shelter to prevent hypo‐ or hyperthermia, respectively. Ungulate species at high‐latitudes select for thermal shelters against cold winds at the cost of forage quality during warmer times (van Beest *et al.* 2012; Street *et al.* 2016; Mason *et al.* 2017). European (van Beest *et al.* 2012) and North American moose (Street *et al.* 2016) prefer mature coniferous forests as thermal shelters over nutritionally more favorable deciduous and open forest habitat. Savanna ungulates seek shade during hot moments of the day thereby reducing foraging time, a reduction that is strongest for larger species (Du Toit & Yetman 2005). However, whether thermoregulatory constraints outweigh foraging constraints or vice versa is context‐dependent. For example, the habitat selection of North American elk (*Cervus elaphus*) in a high‐elevation desert environment was driven more by thermoregulation than food, while in a forest environment, where thermal costs were generally lower, access to food of sufficient quality was the main limiting factor (Long *et al.* 2014). The same study also highlighted within population differences, with individuals that showed the poorest condition at the end of winter selecting more strongly for thermal shelters during spring and summer. Interestingly, these individuals did not increase selection for habitats with higher food quality. This supports the idea that thermoregulatory constraints can be a stronger determinant of fitness differences among individuals than limited food quality (Speakman & Król 2010; Long *et al.* 2016).

## The interplay of surface water dependence and thermoregulation

**Figure 4:**
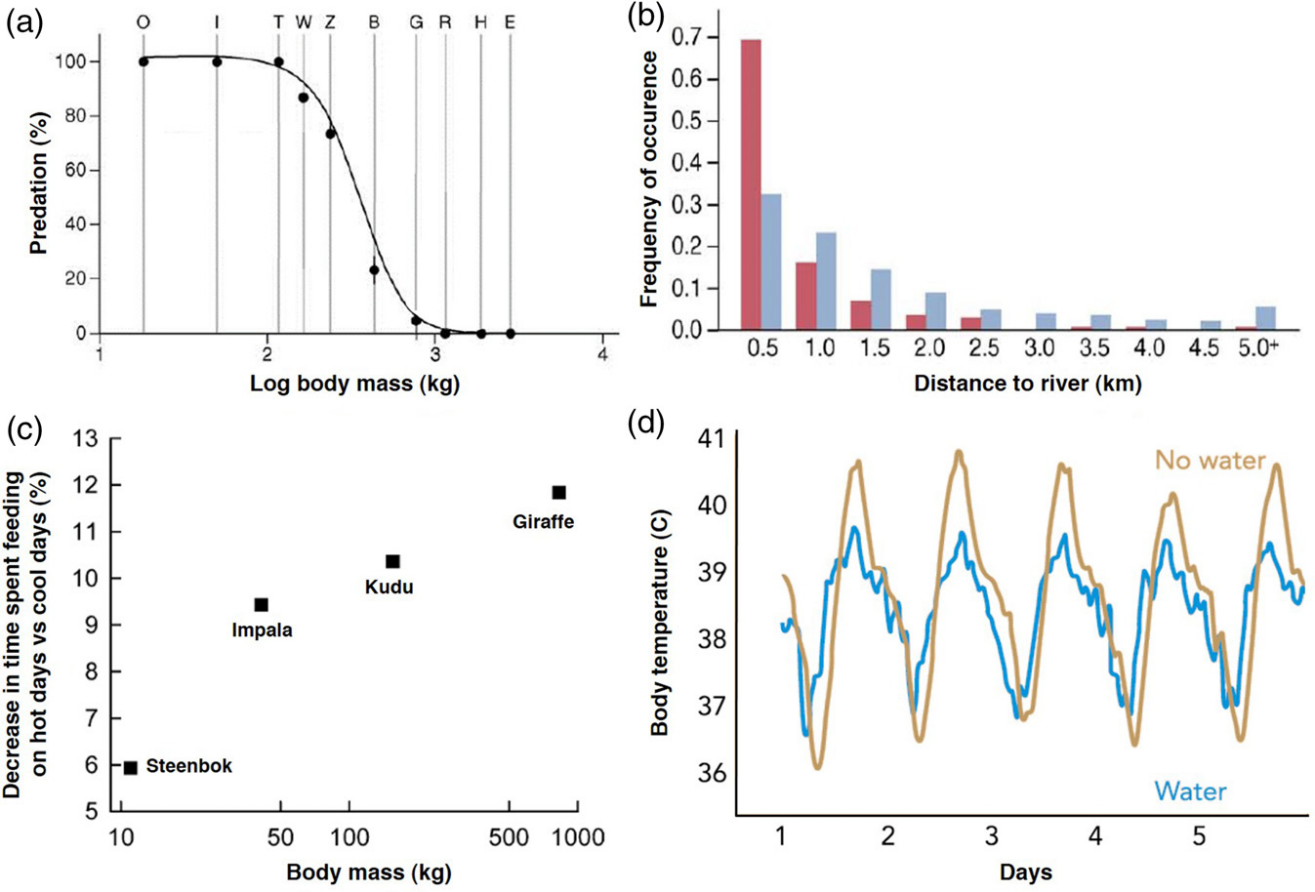
Predation risk and thermoregulation in relation to body size and water availability. (a) Small prey are exposed to more predator species and become increasingly predator regulated. Abbreviations: B = buffalo; E = elephant; G = giraffe; H = hippo; I = impala; O = oribi; R = black rhino; T = topi; W = common wildebeest; Z = plains zebra. (b) Lions select areas that are closer to rivers for hunting more often than expected (red bars), based on the availability of these resources across the landscape (blue bars). (c) Decrease in activity on hot days (max 35 + C) compared to cool days (max 20–24 (c) in terms of percent diurnal time allocated to feeding plotted against body mass for steenbok, impala, greater kudu and giraffe respectively (*R* ^2^ = 0.98; *P* < 0.001) and (d) body temperature of a captive oryx exposed to the same environmental heat but with water (blue line) and a free‐living oryx without water (brown line). Thus, predation risk decreases with increasing body size and distance to river and cooling down is more problematic for larger ungulates with restricted access to water. Reprinted and adapted with permission from (Du Toit & Yetman 2005; Hopcraft *et al.* 2010; Fuller *et al.* 2014).

As outlined previously, body mass is a key trait governing sensitivity to hyperthermia for savanna ungulates (Fig. 4c). However, larger savanna ungulates can compensate for this by accessing water more frequently to cool themselves down (Fig. 4d) suggesting an interplay between surface water dependence and thermoregulation needs. Evaporative cooling can be an important way of losing heat (Tattersall *et al.* 2012) but strongly increases water requirements and is thus extremely costly when drinking water availability is limited. Some extreme drought‐adapted species such as the Arabian oryx (*Oryx leucoryx*) have in fact been found to prioritise the restriction of water loss over maintaining body temperature homeostasis; they tolerate increased body temperature to preserve water (Hetem *et al.* 2016). Furthermore, bathing or wallowing is an important behaviour to cool down but requires the presence of sufficient surface water. *Species that prefer to stay close to permanent rivers or lakes during the dry season (species with high water requirements) are therefore expected to have fewer problems with increasing temperatures as they can increase water intake and use it to compensate* (**H3**; Fig. 3). Indeed, large water‐independent species have specific adaptations to cope with high temperatures such as feeding nocturnally, an elongated shape with large surface area to volume ratio (Mitchell *et al.* 2004) and long legs so that the body is far away from the hot boundary layer close to the ground (Clarke 2017). However, these species would face greater difficulties if droughts and climate warming cause the permanent water bodies or wetlands to substantially shrink or dry out (Crafter *et al.* 1992).

## The interplay of surface water dependence and predation risk

Emerging evidence shows that spatial niche differentiation of species with different water requirements can be mediated by predation risk (Box 2; (Ogutu *et al.* 2014)). This is because concentrations of ungulates near water attract predators that might also benefit from increased cover in catching their prey. Lions (*Panthera leo*) are more commonly found close to water sources (Ogutu & Dublin 2004; Valeix *et al.* 2010) and kill more prey near surface water sources than expected by chance (Hopcraft *et al.* 2005; de Boer *et al.* 2010; Davidson *et al.* 2013). Plains zebras (*Equus quagga*) move away from water sources during nighttime reducing their exposure to hunting lions (Courbin *et al.* 2018). Although still few, these studies suggest that water‐dependent species generally experience higher exposure to predation, especially close to surface water (see also Box 2). Altogether, this suggests that *water‐dependent ungulate species (high minimum dung moisture) generally experience higher exposure to predation because predator densities are higher close to surface water* (**H4A**). Since potential mortality from predation is also inversely related to body size (Fig. 4A), it is expected that *the smaller water‐dependent species are particularly at risk* (**H4B**, Fig. 3). It is now time to upscale species‐specific studies of carnivore–ungulate interactions to community wide investigations (Montgomery *et al.* 2019).

Box 2 **Niche differentiation along the water requirements dimension: surface water–predation interactions in Kruger National Park**

Kruger National Park encompasses a gradient in mean annual rainfall from 750 mm in the south‐west to 450 mm in the north‐east (Venter *et al.* 2003). Between the four perennial rivers that traverse the park, surface water persisted through the dry season only in pools in some of the seasonal rivers and in a few long‐lasting pans or springs. Ungulates concentrate around these water sources and heavily graze in their vicinity, so that much vegetation remains unutilised remote from water. To spread animals more widely and alleviate the intense local forage depletion, the park authority constructed numerous dams, weirs and boreholes in areas that lacked perennial water sources (Smit 2013). Subsequently, zebra (*Equus quagga*) moved from the central region where most grass got consumed into the northern region during the extreme 1982‐3 drought, where more food remained because of low ungulate numbers, formerly constrained by lack of water but now provisioned with artificial water points (Harrington *et al.* 1999). With greater prey availability, lion (*Panthera leo*) numbers also increased in the north. When the next drought occurred in 1986‐7, the rarer antelope species found mostly in the north had to contend with abundant predators as well as little food. Populations of sable antelope (*Hippotragus niger*), roan antelope (*Hippotragus equinus*), tsessebe (*Damaliscus lunatus*) and eland (*Tragelaphus oryx*) crashed (Ogutu & Owen 2003). The increased surface water availability thus benefited especially zebra, with their greater water dependency, to the detriment of overall ungulate diversity in the park. The rare antelope species affected all produce very dry dung pellets, enabling them to survive in areas remote from water, unlike the more common grazers like zebra, buffalo (*Syncerus caffer*) and wildebeest (*Connochaetes taurinus*) (Fig. 3; (Woodall & Skinner 1993)). The effect of excessive surface water provision has been to occlude the spatial heterogeneity that allowed both highly water‐dependent and less water‐dependent ungulates to coexist. The latter benefit especially through occupying areas where predation pressure is reduced because of the lack of the abundant grazers that form the primary prey of lions (Owen‐Smith & Mills 2008).

## Trade‐off between predation risk and thermoregulation

Our integrated framework also suggest a trade‐off between exposure to predation risk and thermal stress that stretches across the two dimensions (food and water requirement) of Fig. 3. As far as we know, this trade‐off has not yet been investigated. Animals temporally adjust their activities to variation in temperature and, during hot periods, become less active or shift from diurnal to nocturnal or crepuscular activity (Du Toit & Yetman 2005; Hetem *et al.* 2012; Owen‐Smith & Goodall 2014). This may increase the risk of being killed by nocturnal predators.

Ungulates can also behaviourally adjust their habitat use by selecting cooler parts of the landscape to prevent heat stress, such as shady and/or breezy areas (Hetem *et al.* 2007; Kinahan *et al.* 2007). Spatial variation in ambient temperature may thus be a strong driver of landscape use by ungulates (Kinahan *et al.* 2007; Bowyer & Kie 2009; van Beest *et al.* 2012; Wiemers *et al.* 2014). In other words, ungulates perceive a ‘landscape of heat' (‘thermal landscape' sensu Sears *et al.* 2016) and in savannas are expected to avoid very hot places, especially when water is limiting. This ‘landscape of heat' phenomenon as a driver of landscape use is conceptually similar to that of a ‘landscape of fear' in response to heterogeneity in predation risk (Laundré *et al.* 2010). Importantly, woody vegetation (shrubs, trees) shapes both the landscapes of heat and fear. Although it may reduce predation risk for some species (Atkins *et al.* 2019), dense woody vegetation generally seems to increase perceived and actual predation risk (Hopcraft *et al.* 2005; Valeix *et al.* 2009; Ford *et al.* 2014; Riginos 2015) and reduces effective heat loads by providing shade from solar radiation (Bader *et al.* 2007; van Beest *et al.* 2012). As outlined above, exposure to predation declines with body mass (Hopcraft *et al.* 2010) and theory predicts that vulnerability to heat stress increases with body mass (Porter & Kearney 2009; Riek & Geiser 2013). While some work on this trade‐off has been done in rodents (Bozinovic *et al.* 2000), empirical data for ungulates showing how different species trade‐off thermal against fear landscapes are largely lacking (but see Wiemers *et al.* 2014).

Overall, this suggests the existence of an ecological trade‐off between predation and thermal tolerance that remains to be tested (Fig. 3) through investigating how ungulates behaviourally adapt to increased temperatures in both the presence and absence of carnivores. We thus predict that *predation risk compromises the behavioural capacity for thermoregulation of especially smaller water‐dependent ungulates by jeopardising their options to nocturnal activity and to remain in areas near water* (**H5**). The largest species should thus adjust their spatio‐temporal patterns more strongly to minimise the effects of high temperatures (while still meeting their high food requirements) (Kinahan *et al.* 2007; Shrestha *et al.* 2014) but can afford to be active at cooler but potentially riskier times (night) and areas (high cover).

## Concluding remarks and future perspectives

The integration of food and water requirements, predation risk and thermoregulatory constraints yields a two‐dimensional framework that generates testable predictions (H1–H5) on the consequences of climate change for community assembly of Africa's ungulates (Fig. 3). They need to negotiate simultaneously a “landscapes of fear”, a “landscape of food”, a “landscape of heat” and a “landscape of water”, where body size and minimal dung moisture content capture important trait dimensions that explain their niche differentiation and coexistence opportunities in such landscapes. This conceptual framework has important implications for biodiversity conservation. For example, previous work predicts highest potential diversity (most coexistence of small to large species) of ungulates at intermediate rainfall and high soil fertility at the regional scale (Olff *et al.* 2002). Our new framework in addition predicts regional ungulate diversity to increase with landscape heterogeneity in distance to water by enabling water‐dependent and water‐independent species to coexist through spatial partitioning of food. Local wildlife and livestock managers cannot change rainfall, but they can influence the distribution of surface water through dams and boreholes. Ecotourism interests often motivate an increase in the number of water points in protected areas, but our framework suggests that this may come at the cost of species diversity, depending on the landscape setting (see Box 2). Also, predation risk is not only expected to mediate niche differentiation along the surface water‐dependence dimension, but also to influence daily activity patterns, so the loss or reintroduction of large carnivores will not affect all ungulate species evenly.

We suggest that future research tests the predictions in Fig. 3 and the hypotheses outlined throughout the text (H1–H5). We recommend that investigations of food partitioning between African ungulates include the effects of surface water dependence and tradeoffs between thermoregulation and exposure to predation. So far, physiological investigations of the mechanisms of water balance and thermoregulation are often restricted to a few species. But our framework allows generalizable predictions for ungulate species that lack such detailed investigations. Studies investigating the combined effects of food, water, temperature and predation are highly needed, as these factors concurrently affect the ecological interactions of savanna ungulates.

In summary, we propose that gradients in both food availability and distance to surface water set the scene for niche differentiation among savanna ungulates and that thermoregulation and predation risk are related to both niche axes but in opposing ways. We identified key functional traits that integrate constraints from food (body size) and water requirements (minimum dung moisture content) that are easy to measure. It is now time to study the interactions between different constraints and upscale from species specific to community wide investigations. The framework we present here assists in the design of such studies of which the results will aid the anticipation of the consequences to large ungulates of human‐induced global change.

## Glossary

Allometry: the study of the relation of body size to physiology, morphology and behaviour

Ecological niche: an ‘n‐dimensional hypervolume', where the dimensions are environmental conditions and resources, that define the requirements of an individual or a species to practice its way of life, more particularly, for its population to persist.

(Functional) traits: qualities of organisms that define species in terms of their ecological roles

Hyperthermia: an abnormally high body temperature due to failed thermoregulation that occurs when a body produces or absorbs more heat than it dissipates.

Loop of Henlé: a long, U‐shaped portion of the tubule that conducts urine within each nephron in the kidney

Metabolic water: water created inside living organisms through metabolism, by oxidising energy‐containing substances in their food

Nephrons: the microscopic structural and functional units of the kidney.

Niche differentiation: the process by which natural selection drives competing species into different patterns of resource use.

Spiral colon: in contrast to humans, where the descending colon is short and straight, the descending colon of ungulates coils down in a long spiral.

Ungulates: hoofed mammals of the orders Perissodactyla, Artiodactyla, Hyracoidea and Proboscidea.

Urine osmolality: the number of dissolved particles per unit of water in the urine.

## Authorship

M.P.V. led all aspects of the study; M.P.V. and E.S.K. contributed and analysed the data to test the second dimension within the framework; all co‐authors contributed to the development of conceptual ideas and to writing the manuscript.

## Data Availability

All data has already been published and no new data is provided.
