# Supplementary material for: Large herbivore assemblages in a changing climate: incorporating water dependence and thermoregulation
Source: Ecol Lett. 2019 Jul 22;22(10):1536–46. doi: 10.1111/ele.13350 (PMC6851681; doi:10.1111/ele.13350)
Supplement: Supplementary file 1 [file ELE-22-1536-s001.docx]

Supplemental information

Large herbivore assemblages in a changing climate: incorporating water dependence and thermoregulation

M.P. Veldhuis (1,2,3), E. S. Kihwele (1,4), J.P.G.M. Cromsigt (5,6,7), J.O.  Ogutu (8),  J. G. C. Hopcraft (9), N. Owen-Smith (10) and H. Olff (1)

1. University of Groningen, Nijenborg 7, 9747AG Groningen, The Netherlands
2. Princeton University, 106A Guyot Ln, Princeton, NJ 08544, USA
3. Leiden University, Einsteinweg 2, 2333CC Leiden, The Netherlands
4. Tanzania National Parks, Arusha, Tanzania
5. Department of Wildlife, Fish and Environmental Studies, Swedish University of Agricultural Sciences, Umeå, 901 83, Sweden
6. Centre for African Conservation Ecology, Department of Zoology, Nelson Mandela University, PO Box 77000, Port Elizabeth, 6031, South Africa
7. Environmental Sciences group, Copernicus Institute of Sustainable Development, Utrecht University, PO Box 80115, 3508 TC Utrecht, The Netherlands
8. University of Hohenheim, Institute of Crop Science, Biostatistics Unit, Fruwirthstrasse 23, 70599 Stuttgart, Germany
9. University of Glasgow, Glasgow, G128QQ, United Kingdom
10. Centre for African Ecology, School of Animal, Plant and Environmental Sciences, University of the Witwatersrand, Wits 2050, South Africa

Correspondence: Veldhuis, M.P. (m.p.velduis@gmail.com)

Table S1. Species names, abbreviations and body mass.

| NR | SCIENTIFIC NAME | COMMON NAME | ABBREVIATION | MEAN FEMALE BODYMASS (KG) |
| --- | --- | --- | --- | --- |
| 1 | Aepyceros melampus | Impala | IMP | 50 |
| 2 | Alcelaphus buselaphus | Hartebeest | HAR | 150.5 |
| 3 | Antidorcas marsupialis | Springbok | SPR | 31.5 |
| 4 | Bos indicus | Zebu Cattle | CAT | 475 |
| 5 | Camelus dromedarius | Camel | CAM | 800 |
| 6 | Capra hircus | Turkana Goat | GOA | 33.5 |
| 7 | Cephalophus dorsalis | Bay Duiker | BADUI | 19.5 |
| 8 | Cephalophus natalensis | Natal Red Duiker | NDUI | 13 |
| 9 | Cephalophus silvicultor | Yellow-backed Duiker | YDUI | 62.5 |
| 10 | Connochaetes gnou | Black wildebeest | BWIL | 135 |
| 11 | Connochaetes taurinus | Common Wildebeest | WIL | 200 |
| 12 | Damaliscus pygargus | Blesbok | BLE | 62.5 |
| 13 | Diceros bicornis | Black rhinoceros | BRHI | 1050 |
| 14 | Equus asinus | Donkey | DON | 165 |
| 15 | Equus quagga | Plains zebra | PZEB | 212.5 |
| 16 | Giraffa camelopardalis | Giraffe | GIR | 815 |
| 17 | Hippopotamus amphibius | Hippopotamus | HIP | 1505 |
| 18 | Kobus ellipsiprymnus | Waterbuck | WAT | 180 |
| 19 | Loxodonta africana | African elephant | ELE | 2850 |
| 20 | Madoqua kirkii | Kirk's Dik-dik | KDD | 5.5 |
| 21 | Oreotragus oreotragus | Klipspringer | KLI | 13 |
| 22 | Oryx gazella | Gemsbok | GEM | 202.5 |
| 23 | Ovis aries | Fat-tailed Sheep | SHE | 50 |
| 24 | Phacochoerus africanus | Common warthog | WAR | 60 |
| 25 | Philantomba maxwellii | Maxwell's Duiker | MDUI | 8 |
| 26 | Philantomba monticola | Blue Duiker | BUDUI | 6.25 |
| 27 | Raphicerus campestris | Steenbok | STE | 11.5 |
| 28 | Redunca arundinum | Southern Reedbuck | SREE | 67.5 |
| 29 | Redunca fulvorufula | Mountain Reedbuck | MREE | 27 |
| 30 | Sylvicapra grimmia | Common Duiker | CDUI | 18.75 |
| 31 | Syncerus caffer | African Buffalo | BUF | 550 |
| 32 | Tragelaphus angasii | Nyala | NYA | 76 |
| 33 | Tragelaphus oryx | Common Eland | CELA | 450 |
| 34 | Tragelaphus scriptus | Bushbuck | BUS | 42 |
| 35 | Tragelaphus strepsiceros | Greater Kudu | GKUD | 167.5 |
